# Native Valve Endocarditis Caused by Nocardia asteroides in an Immunocompetent Host: A Case Report and a Review of the Literature

**DOI:** 10.7759/cureus.35977

**Published:** 2023-03-10

**Authors:** Gina S Gilderman, Mahmoud Morsy, Nishaal Antony

**Affiliations:** 1 Department of Medicine, Burrell College of Osteopathic Medicine, New Mexico, USA; 2 Department of Medicine, Mayo Clinic, Arizona, USA; 3 Department of Medicine, Burrell College of Osteopathic Medicine, Las Cruces, USA; 4 Infectious Diseases, MountainView Regional Medical Center, Las Cruces, USA

**Keywords:** nocardia in immunocompetent, native valve, immunocompetent, infective endocarditis, nocardia asteroides

## Abstract

*Nocardia* species are a rare cause of infective endocarditis (IE). We describe a case of native valve endocarditis caused by *Nocardia asteroides* in a 38-year-old Hispanic male with no apparent environmental exposures or risk factors for IE. Transesophageal echocardiography revealed severe mitral regurgitation, prompting emergent replacement of the valve. *Nocardia asteroides *were isolated from the tissue culture of the mitral valve. MRI of the brain also demonstrated innumerable micronodular intra-axial lesions throughout the brain, consistent with disseminated nocardiosis. The patient was treated with intravenous trimethoprim/sulfamethoxazole, meropenem, and amikacin for a six-week course, followed by oral trimethoprim/sulfamethoxazole and minocycline for 12 months. Follow-up after 18 months revealed no evidence of relapse. Although several cases of endocarditis due to *Nocardia asteroides *have been reported in immunocompromised hosts, to the best of our knowledge we believe the present case is the first to describe native valve endocarditis by *Nocardia asteroides* in an immunocompetent host with no apparent risk factors for IE.

## Introduction

*Nocardia *spp is a Gram-positive, aerobic, filamentous branching rod that is part of the Actinomycetales family. An environmental pathogen that is found in soil, water, and organic matter, most cases of nocardiosis present with symptoms of cough, bloody sputum production, fevers, chills, weakness, chest pain, loss of appetite, and dyspnea which can evolve into pneumonia, occult abscess formation and in rare cases endocarditis [1-8]. Typically introduced via inhalation, *Nocardia* spp can spread hematogenously to different organs, including the heart causing infective endocarditis [9-13]. Infective endocarditis (IE) is a rare infection affecting the endocardial surface or valves of the heart with high rates of morbidity and mortality [1]. It is most often found in patients with pre-existing structural heart disease; typical pathogens of IE that account for 80-90% of all cases include *Staphylococcus aureus*, *Coagulase-negative Staphylococci*, *Streptococci*, and *Enterococci* [6,7]. In contrast, IE due to *Nocardia* is rare. Patients diagnosed with nocardial endocarditis tend to have underlying comorbidities including immunocompromised state, chronic hemodialysis, transplant recipients, prosthetic heart valves, and intravenous drug use with approximately one-third of patients being immunocompetent; native valve endocarditis is uncommon with a rising incidence of two to 10 cases per 100,000 patients per year [2,3,10]. The literature describing cardiac manifestations of nocardiosis is limited, and rarely has it been reported to affect native valves, let alone in immunocompetent hosts. Here, we report a case of native valve endocarditis caused by *Nocardia asteroides *in an immunocompetent host and provide a review of previous literature regarding *Nocardial* endocarditis.

## Case presentation

**Figure 1:**
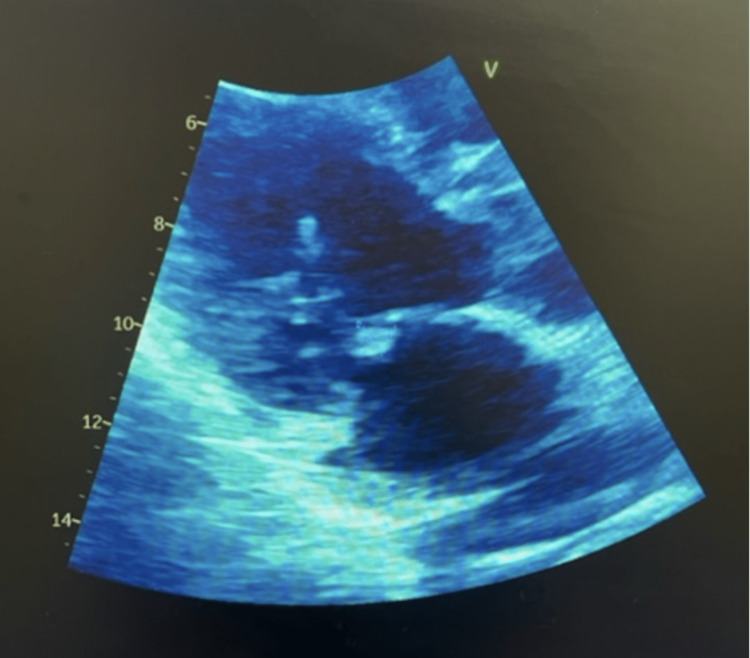
Still from transesophageal echocardiography demonstrating mitral valve vegetations.

A 38-year-old Hispanic male with a history of well-controlled insulin-dependent diabetes mellitus, obstructive sleep apnea, dyslipidemia, and seizure disorder with a spinal stimulator presented to the emergency department with left upper quadrant pain and recurrent fevers for two weeks. He was previously admitted to the hospital nine weeks prior for similar symptoms; however, the previous work-up was unremarkable, and his fevers defervesced following treatment with empiric piperacillin-tazobactam and vancomycin for four days. Previous imaging studies, including a 2D echocardiogram and CT scan of the abdomen and pelvis, were unrevealing with the exception of splenomegaly and he was discharged to home without antibiotics. Upon readmission, he was febrile but did not report chest pain or shortness of breath. Physical examination upon initial consultation was unremarkable with the exception of left upper quadrant tenderness. Blood tests revealed leukocytosis with liver and kidney function within reference ranges. Empirical antibiotic treatment was initiated with cefepime. An initial transthoracic echocardiogram revealed a hypoechoic lesion attached to the anterior mitral leaflet measuring 0.6 x 0.7 cm^2^ in diameter. These changes were not present on his last echocardiogram. A transesophageal echocardiogram demonstrated extensive echogenic changes noted from both papillary muscles of the mitral valve (MV) extending through the chordae to the leaflets (Figure 1).

The average size was noted to be 2.1 x 1.4 cm^2^ in diameter from the posterior medial papillary muscle and 0.9 x 1 cm extending into the leaflets. Findings were concerning for pseudoaneurysm and abscess formation. Multiple sets of blood cultures were negative. These findings were suggestive of culture-negative native valve endocarditis, and the patient remained on empiric cefepime which had been started upon admission to the hospital. Due to the extensive echogenicity of the lesion and mass effect besides severe destruction of the mitral valve leading to acute symptomatic regurgitation, the patient underwent emergent MV replacement. Brain MRI with and without contrast performed 19 days after admission due to reports of confusion demonstrated diffuse tiny enhancing foci located primarily on the right frontal lobe, precentral gyrus, right thalamus, right caudate nucleus, and the ventricular white matter demonstrated T2 hyperintensities. Pathology from the valve demonstrated moderate growth of Gram-positive, long filamentous beaded bacilli, suggestive of *Nocardia* and later confirmed to be *Nocardia asteroides*. He was subsequently started on a triple-drug regimen consisting of trimethoprim-sulfamethoxazole, meropenem, and amikacin. A repeat MRI one month later showed interval progression in a number of innumerable micronodular siderotic intra-axial lesions (size ranging from 2 mm to 5 mm), scattered within the internal and external watershed territories of the bilateral cerebral and cerebellar hemispheres, basal ganglia, and thalami; approximately 50% of the lesions showed enhancement after contrast administration with no evidence of rim-enhancing lesions. Given the extensive nature of the disease, the patient completed a 6-week course of triple-drug therapy with intravenous trimethoprim-sulfamethoxazole, meropenem, and amikacin throughout his hospital stay. Following the completion of intravenous medications, he was discharged and transitioned to oral trimethoprim-sulfamethoxazole and minocycline for 12 months of outpatient suppressive therapy. His follow-up blood work remained unremarkable and the patient completely recovered. Follow-up imaging was not available as the patient transferred care.

## Discussion

Methods

A systemic review of the English language literature was performed to identify cases of *Nocardia* endocarditis using keywords including “native valve,” “infective endocarditis”, “*Nocardia asteroides*”, and “immunocompetent,” searched alone or in combination using the National Center for Biotechnology Information (NCBI) PubMed database. Data collected included patient demographics, specific *Nocardia* *spp*, type of (native; prosthetic) and the affected valve, culture-positive specimen, underlying comorbid conditions that drive immune status, and the presence (or absence) of embolic phenomena. In addition, disseminated nocardiosis was defined as having more than at least one positive blood culture for *Nocardia spp* and/or one non-contiguous organ involved. To date, there have only been 31 cases of nocardial endocarditis reported since 1973; 19 cases involved native valves, while the remaining 12 cases involved prosthetic valves [6,8]. 

Discussion

This is a patient who initially presented with recurrent fevers of unclear origin and evidence of endocarditis. Of the six previously reported cases of *Nocardia* *asteroides* native valve endocarditis, all six occurred in patients of an immunocompromised state. These included renal transplant, steroid therapy, intravenous drug use, malignancy, autoimmune disease, recurrent skin infections, and pre-existing structural heart abnormalities. 

A major risk factor for *Nocardia* infection is impaired T-cell mediated immunity such as in those with human immunodeficiency virus (HIV) or acquired immunodeficiency syndrome (AIDS), chronic steroid use, malignancy, autoimmune disorders, and bone marrow or solid organ transplantation [10,14,15]. A study by Steinbrink et al. 2018 found that dissemination of nocardial infection occurred more often in the immunocompromised arm as nocardiosis is considered an opportunistic infection due to decreased cell-mediated immunity [10,11,16]. Although *Nocardia* infections occur primarily in immunocompromised hosts, approximately one-third of reported cases are in immunocompetent subjects [17,18, 19,20]. Underlying comorbidities, such as chronic obstructive pulmonary disease (COPD) or bronchiectasis, may also predispose immunocompetent patients to infection. However, this area is not well-established at the present time. There have been several reports of healthy patients experiencing “silent” dissemination of *Nocardia* to the CNS in the absence of specific symptoms that was only discovered by MRI study [10,16,17,19-22]. Anagnostou et al. 2014 found that, while CNS nocardiosis could occur in both immunocompetent and immunocompromised populations, dissemination to the CNS could be the result of either a pulmonary infection or could exist on its own without the presence of even endocarditis [17]. Whether these patients had a defect in their host responses or an undiagnosed or untreated immunocompromising condition is possible, but not known. 

The presentation of nocardiosis, including nocardial endocarditis, is highly variable which may lead to a delay in diagnostic testing and initiation of treatment. Diagnostic imaging should be tailored to the presenting symptoms. Given predilection for CNS involvement, it is recommended in all patients with nocardial infection perform brain imaging [17,18]. Further, we propose that it may also be reasonable to perform echocardiography in disseminated cases, particularly because mortality by nocardial infection is roughly 40% [23-26]. Treatment of *Nocardia* infection is with sulfonamide drugs such as trimethoprim-sulfamethoxazole, and other antibiotics such as amikacin and imipenem have been used as effective agents against nocardiosis as they have demonstrated synergistic effects when combined with trimethoprim-sulfamethoxazole [22, 27]. Duration of treatment is typically months to greater than one year with multiple agents due to a poor response to monotherapy. Further, recurrent (or relapsing) infection is not uncommon with a rate of approximately 5% in immunocompromised individuals [17,28]. Recurrence may occur within months to years after primary infection, hence why close follow-up for at least one year following completion of antimicrobial therapy is necessary. A review of the literature spanning the last 40 years regarding *Nocardia* complicated with endocarditis was performed and is outlined below (Table 1).

**Table 1:** A review of literature for Nocardia species endocarditis

Author\Year	Patient Demographic	Specific Nocardia spp	Valve Type	Culture Positive Specimen	Comorbid conditions	Embolic phenomenon	Antibiotics used	Valve Surgery	Survival
Njie et al., 2021 [29]	34/F	Nocardia nova	Mitral Valve (native)	4 out of 4 blood cultures	Chronic Lyme disease Indwelling peripherally inserted central catheter	Septic Pulmonary emboli	Imipenem TMP/SMX + Amikacin Imipenem + Amikacin Ceftriaxone	No	Survived
Enwezor et al., 2021 [30]	54/M	Nocardia Spp.	Aortic Valve (native)	2 out of 4 blood cultures.	Hypertension, Alcohol abuse, Injection drug use, and HBV infection.	White matter infarcts, splenic infarcts	Vancomycin + Cefepime TMP/SMX + amikacin + Imipenem/Cilastatin	No	Died
Kiyasu et al., 2021 [6].	22/F	Nocardia Nova.	Vegetation on the posterior papillary muscle of the left ventricle	2 out of 3 blood cultures	Adult-Onset Still’s Disease. Treated by Prednisolone and cyclosporin	aneurysm of the right middle cerebral artery	Moxifloxacin (7d) Moxifloxacin (5d) Cefazolin (2d) Tazobactam\piperacillin + Daptomycin (4d) Daptomycin + Imipenem/Cilastatin + Amikacin Imipenem + Amikacin (~80d) TMP/SMX ~1Y	No	Survived
Gupta et al., 2019 [31]	53/F	Nocardia Spp.	Aortic Valve (native)	3 out of 3 blood cultures	History of Breast cancer treated with surgery, chemotherapy, and immunotherapy completed 3 months before symptom onset	Lung Parenchymal nodules.	Meropenem + Amikacin (8w) Linezolid (4.5m)	Yes	Survived
Jackson et al., 2018 [32]	39/M	Nocardia Farcinica.	Tricuspid Valve (native)	Blood cultures, cultures from percutaneous drains	Post splenectomy, Intravenous drug use	Both adrenal glands	TMP/SMX + meropenem (4w) TMP/SMx	No	Lost to follow up
Hazim et al., 2018 [9].	64/M	Nocardia Asteroides.	Tricuspid Valve (transplanted heart)	Blood cultures	Transplanted heart recipient on Tacrolimus, Prednisone, and mycophenolate	Abscess within the right vastus lateralis Ring enhancing lesion in the brain	TMP/SMX + Imipenem/Cilastatin TMP/SMX +Ceftriaxone Linezolid + Ceftriaxone Linezolid + Minocycline Linezolid	No	Survived
Delvenne et al., 2017 [33]	55/M	Nocardia Farcinica.	Aortic Valve (native)	Pus in the brain	Systemic sclerosis on mycophenolate mofetil, methylprednisolone	Brain	Ceftriaxone + TMP/SMX (4w) Imipenem + TMP/SMX (8w) TMP/SMX (9m)	No	survived
Majeed et al., 2017 [34]	72/M	Nocardia Kroppenstedtii.	Mitral Valve (native)	Blood cultures	Mantle Cell lymphoma on rituximab, bendamustine bortezomib	Brain	Meropenem +TMP/SMX Levofloxacin + linezolid Ceftrixone + minocycline + ciprofloxacin	No	Died
Kuretski et al., 2016 [35]	58/F	Nocardia Farcinica.	Mitral Valve (native)	3 out 4 blood cultures Pathology of the resected valve	Obstructive Pulmonary Disease oral cancer status after resection Patent foramen ovale after correction	ND	Rociphen (2w) Meropenem + Amikacin (2w) Ciprofloxacin	Yes	Survived
Bianchi et al. 2011 [36]	36/M	Nocardia Spp.	Mitral Valve (native)	Vegetations on explanted mitral valve	Liver Transplant Recipient. On Cyclosporine, Azathioprine, and Hydrocortisone	No available data	Cefaclor (10d) Ceftriaxone then Vancomycin TMP/SMX	Yes	Survived
Cargill et al., 2009 [37]	85/F	Nocardia Cyriacigeorgica.	Mitral Valve (native)	Pus from thigh and chest wall abscesses	Ischemic Heart Disease, Chronic Obstructive Pulmonary Disease, Poly Myalgia Rheumatica on prednisone	Thigh, forearm, and chest wall abscesses	Doxycycline	No	Died
Daniel et al., 2007 [38]	22/F	Nocardia Farcinica.	Left ventricular wall	Blood cultures	Lupus nephritis on prednisone and cyclophosphamide	Ring enhancing nodules in Subcutaneous tissues, paraspinal muscles, kidneys, brain, and right lung	TMP/SMX +Ciprofloxacin + imipenem (3wks) Amikacin (2wks) TMP/SMX (12m)	No	Survived
Antony et al., 2006 [18]	74/F	Nocardia Asteroides.	Mitral Valve (native)	Replaced Valve, blood cultures	Hypertension, Valvular Heart Disease	None	vancomycin + gentamicin imipenem + ceftriaxone (6w) TMP/SMX (6m)	Yes	Survived
Breitkopf et al., 2005 [39]	33/M	Nocardia Paucivorans.	Mitral Valve (native)	Blood, resected valve	No available data	No available data	No available data	No available data	No available data
Lazo Torres et al., 2004 [22]	53/F	Nocardia Spp.	Mitral Valve (native)	Resected valve	None (history of tooth extraction 3 days prior)	No available data	Imipenem + Amikacin (4wks) TMP/SMX (6m)	Yes	Survived
Watson et al., 2001 [40]	39/M	Nocardia Asteroides.	Aortic Valve (native)	Blood culture, ulcer swab, replaced valve specimen	intravenous drug user	Toe	Meropenem + Amikacin + Ceftriaxone + TMP/SMX Ceftrixone + TMP/SMX Cefpodoxime + TMP/SMX TMP/SMX (6m)	Yes	Survived
Dhawan et al., 1998 [41]	46/F	Nocardia Spp.	Mitral Valve (native)	(none) Histology of the Valve specimen. Gram Staining of ankle specimen	Flial Mitral Valve, Recurrent skin infections. But no evidence of immunosuppressive disease.	Toe	TMP/SMX + Imipenem/Cilastatin (1m) TMP/SMx (9m)	yes	Survived
Niehues et al., 1996 [42]	62/M	Nocardia Asteroides.	Aortic Valve (native)	Excised thigh nodule	Kidney transplant on methylprednisolone, cyclosporine A	Eyes, lung, extremities, brain	Imipenem/cilastatin + Doxycycline	No	Died
Leonard et al., 1973 [43]	34/F	Nocardia Asteroides.	Aortic Valve + Mitral Valve (native)	No available data	Renal Transplant on Steroids	Brain	No Available Data	No	Died

## Conclusions

We described the first case of native valve endocarditis caused by *Nocardia* *asteroides* in an immunocompetent host. Although endocardial involvement in *Nocardia* *spp* is uncommon, it is accompanied by high morbidity and mortality and should be considered during work-up in culture-negative endocarditis. 
